# Dosimetric validation of first helical tomotherapy Hi-Art II machine in India

**DOI:** 10.4103/0971-6203.48717

**Published:** 2009

**Authors:** Rajesh A. Kinhikar, Swamidas V. Jamema, Reenadevi, Rajeshri Pai, Master Zubin, Tejpal Gupta, Deepak S. Dhote, Deepak D. Deshpande, Shyam K. Shrivastava, Rajiv Sarin

**Affiliations:** 1 Department of Medical Physics, Tata Memorial Hospital, Parel, Mumbai, ^2^Department of Radiation Oncology, Tata Memorial Hospital, Parel, Mumbai, India; 3 Department of Advanced Centre for Treatment, Research and Education in Cancer (ACTREC), Kharghar, Navi Mumbai, India; 4 Department of Brijlal Biyani Science College, Amravati, India

**Keywords:** Tomotherapy, commissioning and acceptance, dosimetric validation

## Abstract

A Helical Tomotherapy (HT) Hi-Art II machine, Hi ART (TomoTherapy, Inc., Madison, WI, USA) was installed at our center in July 2007, and was the first machine in India. Image-guided HT is a new modality for delivering intensity modulated radiotherapy (IMRT). Dosimetric tests done include (a) primary beam alignment (b) secondary beam alignment (c) water tank measurements (profiles and depth doses) (d) dose rate measurements (e) IMRT verification, and (f) Mega voltage Computed Tomography (MVCT) dose. Primary and secondary beam alignment revealed an acceptable linear accelerator (linac) alignment in both X and Y axes. In addition, it was observed that the beam was aligned in the same plane as gantry and the jaws were not twisted with respect to gantry. The rotational beam stability was acceptable. Multi-leaf collimators (MLC) were found to be stable and properly aligned with the radiation plane. The jaw alignment during gantry rotation was satisfactory. Transverse and longitudinal profiles were in good agreement with the “Gold” standard. During IMRT verification, the variation between the measured and calculated dose for a particular plan at the central and off-axis was found to be within 2% and 1mm in position, respectively. The dose delivered during the TomoImage scan was found to be 2.57 cGy. The Helical Tomotherapy system is mechanically stable and found to be acceptable for clinical treatment. It is recommended that the output of the machine should be measured on a daily basis to monitor the fluctuations in output.

## Introduction

Intensity modulated radiotherapy (IMRT) has been a major paradigm shift in cancer management and its clinical application is still evolving. Helical Tomotherapy^TM^ (HT) Hi-Art II (TomoTherapy, Inc., Madison, WI, USA) is one of the important innovations in clinical applications that has become feasible as a consequence of technology development in Image-guided HT, and represents a new form of radiation treatment delivery with IMRT. A 6 MV linear accelerator (LINAC,) is mounted on a ring gantry that continuously rotates while the treatment couch is translated along the axis of gantry rotation during treatment delivery. A 64-leaf binary collimator is used to subdivide the fan beam into beamlets. Intensity modulation (IM) is thus achieved by temporal modulation of the collimator leaves.

**Figure 1:**
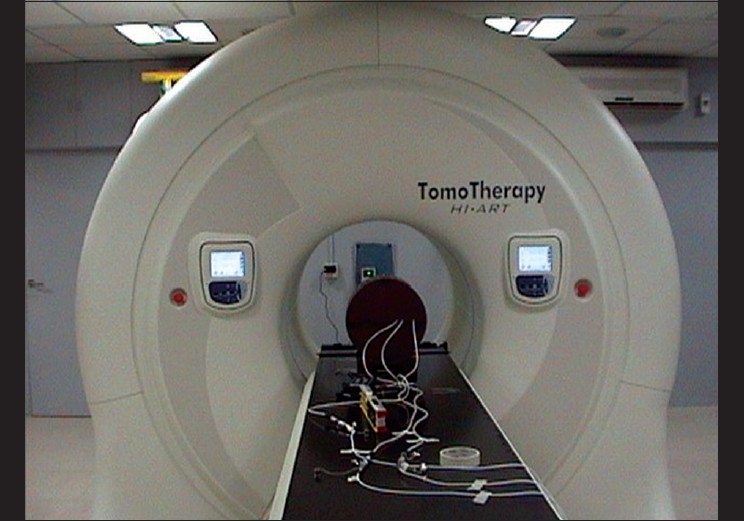
Helical tomotherapy machine

The HT machine [Figure 1] was installed at our center in July 2007, and was the first machine in India. The complex design[1–6] of the unit requires high-end mechanical control and extreme synchronization, to modulate radiation beam intensity. Thus said, an extensive mechanical and dosimetric verification is required.

The purpose of this study is to report the results obtained from the acceptance test procedures[7] (ATP) and dosimetric tests[8] carried out during installation of the HT system at our center. The objective and significance of each test is also discussed.

## Materials and Methods

### Primary beam alignment

#### Alignment in the X direction (Tongue and Groove Procedure)

The exact positioning of the radiation source is critical, due to narrow beamlets and the relatively short distance from the source to the primary collimator. The purpose of this test is to verify whether the source is centered in the X direction with respect to the MLC, and if the source and MLC are stable as the gantry rotates. Placing the source in a position such that it is aligned to the center of the MLC ensures profile symmetry and consistency in the International Electrotechnical Commission (IEC) X direction.

To accomplish this test, the “rotational tongue and groove procedure (T and G)” was used. Radiation was delivered (with gantry at 0^°^) wherein every even leaf was open with all odd leaves closed, followed by every odd leaf open with all even leaves closed. This produced a series of T and G modulations. This procedure was delivered with the couch fully retracted from the bore. The onboard MVCT detector data was used to analyze the T and G results. The xenon detector recorded the incident dose profile. The profile was visually checked for its symmetric pattern. In addition, the “Net Percent out of Focus” and “Linac shift” were also calculated from the profile.

#### Alignment in the Y direction (Jaw Shift Procedure)

The purpose of this test was to verify that the source was centered in the Y direction with respect to the jaws. The “Jaw shift procedure” was used to check the source alignment in the Y-axis. An A17 ion chamber (Standard Imaging, Middleton, WI, USA) was placed on the patient table. It was a cylindrical ion chamber with a 12cm-long collecting length and 8mm diameter. The long axis of the ion chamber was positioned parallel to the Y axis, at the isocenter. The 4mm Y-axis field width struck the chamber at different inferior–superior regions as a function of the moveable-jaw shift position, but the detected signal was approximately invariant with an ion chamber position, due to its uniform response. The central 5cm of this chamber was irradiated as the jaws were shifted from −20 to 20mm. The chamber response in this region was constant enough, within 1%, such that each individual reading could be used without correction. This measurement was forgiven for small translation setup errors in any direction. The charge collected by the ion chamber was recorded by an eight-channel Tomo Electrometer supplied by the manufacturer.

#### Central axis Y-axis misalignment

The purpose of this test was to verify that the beam was aligned in the same plane as the gantry, so that the jaws were not twisted with respect to the gantry, which means, the beam should be parallel to, and directed, in the plane of gantry rotation. A 1cm slab of solid water (Gammex Inc, Middleton, WI, USA) was placed on the patient table in such a way that it was centered under the overhead (virtual isocenter) laser. An extended dose range (EDR2, Eastman Kodak, Rochester, NY) film (35cm × 43cm) was placed diagonally on the solid water slab so the maximum width of the beam could be captured on the film. A 1cm slab of solid water was placed over the film. The film was placed 23cm below the isocenter. The film was irradiated through all leaves on one side of the MLC, 1-32, with the gantry at 0°. The moveable jaws defined a 2cm-long Y-axis field width. The gantry was then rotated 180° and the exposures were repeated using the same open-leaf configuration. Opening half of the MLC leaves for both exposures resulted in abutting images, which did not significantly overlap at the beam center. The film was analyzed, and the center of each half-field exposure was independently determined and compared.

#### Rotational beam stability

This test was a constancy (output and energy) quality assurance check. The purpose of this test was to evaluate the performance of the linac output and energy with the help of an on-board MVCT detector array. Rotational treatment was delivered with all leaves open, a jaw width of 1cm, and the couch retracted out of the bore. The MVCT detector captured the shape of the lateral beam profile at each linac pulse. The data were averaged over the maximum number of rotations that fit in the set. Pulse by pulse Hi-Art ion chamber measurements characterized the variation of output with the gantry angle. The ratio of the measured average profile to a reference profile was obtained and the constancy (output and energy) was estimated.

### Secondary beam alignment

#### MLC Center-of-Rotation (COR) twist

The MLC leaves could have a twist with respect to the plane of gantry rotation, as the case for the jaws, since MLC mounting is independent of the jaws. The alignment could be tested via double exposure, with gantry-rotation delivery to a film placed at the isocenter. The purpose of this test was to ensure that MLC was properly focused toward the source and the MLC was laterally centered above the rotational center of the gantry. An EDR2 (22cm × 25cm) film was placed on the solid water slab at the isocenter with 5cm buildup.

In this procedure, the film was irradiated at the isocenter with leaves 27 and 28, and 32 and 33 open (two adjacent off-axis leaves, and the two center leaves) from 0 degrees. The MLC center was between leaves 32 and 33 so the resultant two leaf-32 images had to be adjacent and not superimposed. The same film was then irradiated from 180 degrees with only leaves 27 and 28 open. This magnified any MLC offset from the gantry isocenter. The offset was then determined by half the difference in the distance between the “leaves 27/28” exposures and the central leaves exposures. Any “twist” of the MLC with respect to the plane of rotation could be determined from this test as well, by simply comparing the edges of the “leaves 27/28” exposures. The MLC twist angle was given by half the difference in the angle between the 0 and 180 degree exposures.

#### Field center versus jaw setting

The purpose of this test was to ensure that the jaws closed symmetrically, so that the centers of the different field sizes were consistent with each other. An EDR2 film (35cm × 43cm) was placed on the solid water slab diagonally for maximum lateral length. Another slab of solid water (5.0cm) was placed on top of the film. The moveable jaw setting was set to the clinical width of 5cm. Leaves 11-18, 29-36, and 47-54 were opened and the film was exposed. Next, the moveable jaws were narrowed to the clinical Y-axis 1cm field width. Leaves 20-27 and 56-63 were then opened, and the film was exposed again. Similarly, the same film was exposed for a clinical jaw width of 1cm. The film was analyzed and the centers of each leaf set were determined from the resulting dose images.

### Water tank measurements (profiles and depth doses)

#### Transverse and longitudinal beam profiles

**Figure 2:**
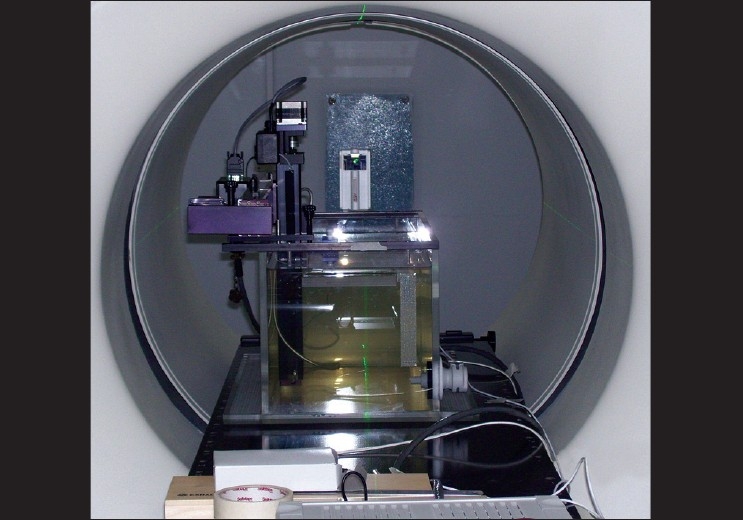
A customized water tank (Dimensions: 45 cm width, 75 cm length, and 30 cm height) supplied by the manufacturer for measurement of profiles and CADD

The purpose of this test was to verify that the beam shape and energy were consistent with the beam model created at the factory, and to ensure the accurate prediction and delivery of the IMRT plans. A customized water tank (Dimensions: 45cm width, 75cm length, and 30cm height) supplied by the manufacturer is shown in [Figure 2]. This water tank had two dimensional movements only (longer direction and the vertical). The A1SL ion chamber was placed into the water tank. The A17 ion chamber, in its buildup cap, was used as a reference chamber and was placed just outside the tank. A procedure was selected with the field width 5cm (all leaves open) and transverse profiles were measured for depths (1.5, 5, 10, and 20cm). The transverse profiles were also obtained for field widths of 2.5 and 1.0cm each.

The tank was then oriented by 90° such that when the ion chamber was at its home position, the centroid of its collection volume coincided with the intersection point of the overhead laser. The longitudinal profiles were obtained for the field width (5, 2.5, and 1cm) at 1.5, 5, 10, and 20cm depths with a static procedure (with 40 central leaves open). The measured transverse and longitudinal profiles were then compared with the factory data. The field width and the gamma (2% and 1mm) were evaluated.

#### Central axis depth dose

With the same setup, the central axis depth doses (CADD) were also measured for the field width (5, 2.5, and 1cm) up to a 20cm depth. For measurements of CADD, a static procedure (with 40 central leaves open) was performed. The depth of dose maximum (D_max_) was estimated. In addition, the depth dose values at 10 and 20cm were compared with the reference data. The ratio (D20/D10) was also calculated and compared with the reference data.

### Dose rate

**Figure 3:**
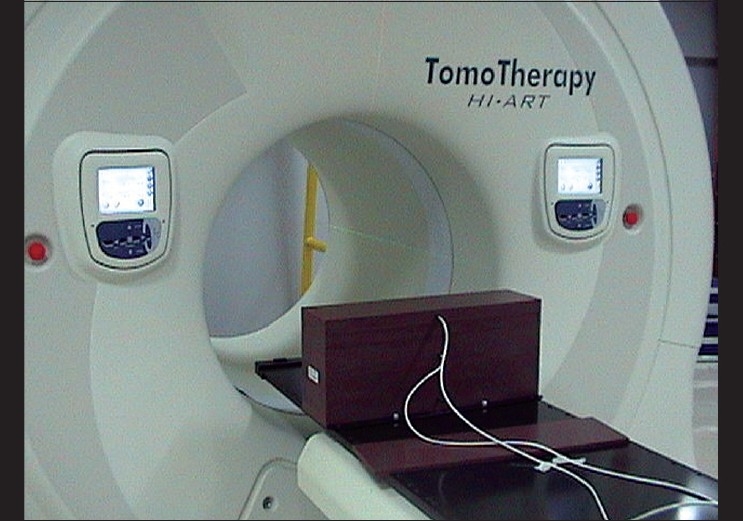
The setup for output measurement in solid water slabs at the isocenter (85 cm)

An A1SL ion chamber was set in solid water phantom slabs for a field size of 5cm × 40cm at an isocenter (85cm), with a buildup of 1.5cm. The setup is shown in [Figure 3]. The central 40 leaves were set open. The charge collected by the ion chamber was recorded with the help of a TomoElectrometer and the dose rate at the isocenter was calculated and compared with the nominal dose rate.

### IMRT verification

**Figure 4:**
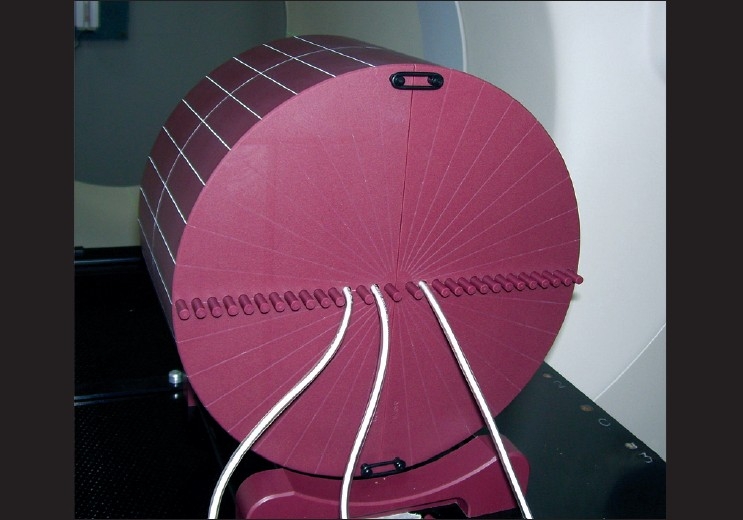
TomoPhantom along with an A1SL ion chamber

The purpose of this test was to verify that a treatment to a phantom, IMRT would be consistent with the values calculated from the beam model by the Tomotherapy planning system. The TomoPhantom with A1SL ion chamber as shown in [Figure 4] was used for this purpose. The appropriate IMRT verification patient/plan in which the treatment region of interest (ROI) lies on central axis or at off-axis was selected. The dose was measured on central axis and at off-axis for the respective IMRT plans for 1, 2.5 and 5cm jaw widths. The charge(s) collected by the electrometer(s) was recorded and the dose was calculated. The measured dose for On-Axis plan and Off-axis plan was then compared with the calculated dose.

### MVCT dose

The purpose of this test was to ensure that dose during MVCT imaging is within factory specifications. The TomoPhantom was used for this purpose. An A1SL ion chamber was inserted in the centre of the TomoPhantom. A procedure with an open field rotating was performed and fifteen images were captured. The charge from the electrometer was recorded and the dose was calculated.

## Results and Discussion

### Primary beam alignment

The primary and secondary beam alignment tests are largely analogous to the testing of conventional linac jaw symmetry and collimator rotation angle. These elements of tomotherapy geometry impact on the treatment delivery in similar ways to their conventional linac. Primary beam adjustment is relatively difficult and hence should be done initially. This is because all the back-shielding lead needs to be removed before the accelerator can be moved. On the other hand, the MLC/source alignment is more easily adjusted by moving the source rather than the MLC. This is achieved with the help of precise servomotors that control the relative motion of the jaws. The adjustments of Y-axis misalignment is possible with a parameter input value which can be controlled by simple software.

#### Alignment in the X direction (Tongue and Groove Procedure)

**Figure 5:**
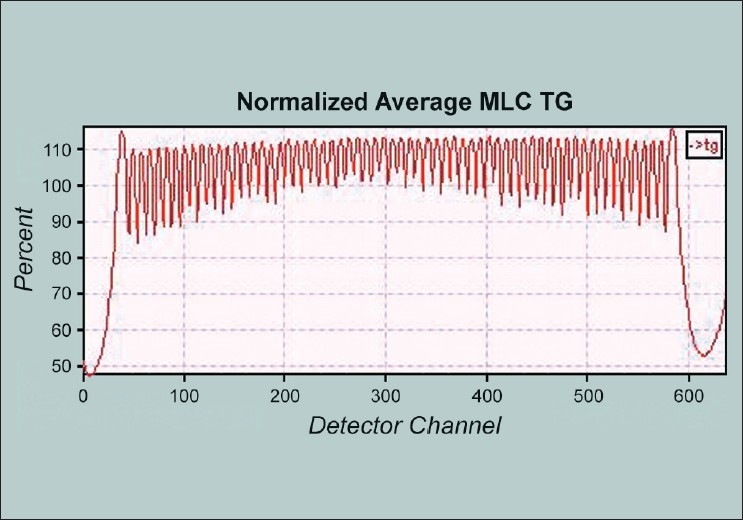
The results of the MLC T and G procedure that revealed acceptable linac × axis alignment. MLC is positioned with respect to the center of rotation and is aligned with respect to the radiation plane. The percent out of focus was found to be 0.38 (specification <2%). The linac shift was found to be 0.026 mm (spec <0.3 mm)

Most of the data was analyzed with the dedicated software programs available only with the manufacturer. [Figure 5] shows the results of MLC TandG procedure that revealed acceptable linac X axis alignment. MLC was positioned with respect to centre of rotation and was aligned with respect to the radiation plane. Percent out of focus was 0.38 (specification <2%). The linac shift was found to be 0.026mm (spec <0.3mm). This test reveals that the source was 0.026 mm away from the center of the primary collimator, after accounting for the magnification of the movement at the isocenter.

The T and G profile can be obtained by film, or by scanning an ion chamber across the all leaves open, odd leaves open, and even leaves open profiles, but it is easier and faster to utilize the onboard MVCT detector to collect the profiles. We used this MVCT detector data. The data were processed such that the all-even-leaf profile was added to all-odd-leaf profile. This determined how well the xenon detectors could differentiate the fluence.

Tomotherapy has written software for facilitating the analysis of film and detector data. Any test that can be performed with the detector data can be automated and analyzed quickly. The detector array proved very useful for analysis. It was able to analyze its consistent mapping with the MLC leaves and its alignment with the gantry rotation plane. It was found that it is capable to adequately replace film for few tests. The spatial resolution of the detectors was almost as fine (0.6 mm) as digitized film results, as determined by the transverse profiles.

#### Alignment in the Y direction (Jaw Shift Procedure)

**Figure 6:**
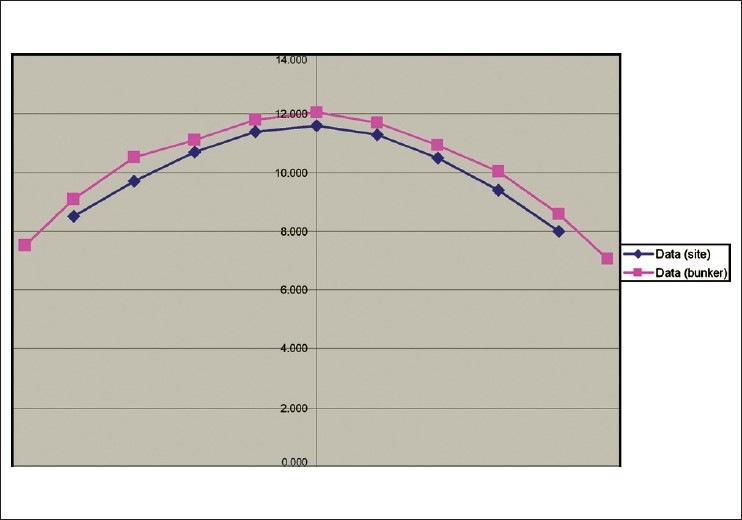
The relative intensity measured as a narrow jaw width. The jaw shift procedure showed acceptable linac Y alignment

Slight misalignment in the Y direction would be magnified and readily noticeable. The moveable jaws pivot about a point that is 5cm behind the target position. Thus SAD is 85cm and the pivot-point to the isocenter distance is 90cm. The moveable jaws were asymmetrically shifted about their nominal center point such that the jaws-defined beam center swept across the physical source position. The moveable jaw offsets were swept from −20 to 20mm at the isocenter plane in a series of discrete positions. All MLC leaves were open for each treatment procedure delivered for each static position of the moveable jaws. The moveable-jaws-defined Y-axis field width was kept constant at a relatively small distance of 4mm projected to the isocenter. [Figure 6] shows the relative intensity measured as a narrow jaw width. Jaw shift procedure showed acceptable linac Y alignment. Suggested Y move was less than 0.3mm.

#### Central axis Y-axis misalignment

**Figure 7:**
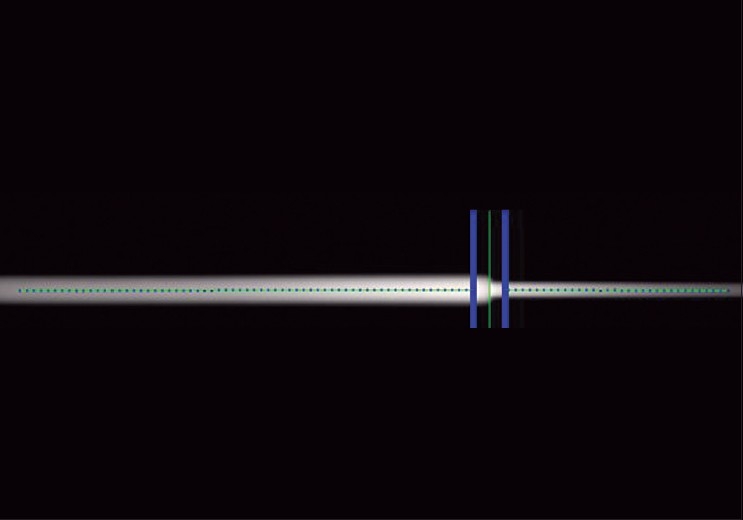
The resultant dose image for the central axis Y-axis misalignment test. The central axis Y-axis misalignment procedure showed acceptable alignment. Y-divergence offset was less than 0.5 mm

Figure 7 shows the resultant dose image for central axis Y-axis misalignment test. Central axis Y-axis misalignment procedure showed acceptable alignment. Y-divergence offset was less than 0.5 mm. Jaw twist angle was found to be less than 0.5°. This test confirmed that the beam was aligned in the same plane as gantry and the jaws are not twisted with respect to gantry.

Any Y-axis misalignment of the beam center could be detected by placing the film well away from the gantry isocenter below the axis of rotation so radiation directed from above had a relatively long distance to travel and therefore to diverge. Next the gantry can be rotated 180° and the film can be irradiated again. The second exposure traveled less before it struck the film, so it diverged less. Any Y-axis misalignment of the beam center away from the gantry plane of rotation was readily apparent on the exposed image because the longitudinal centers of the two exposures were different. The test performed in this study for central axis Y-axis misalignment only verifies that a specific jaw setting does or does not have a beam center with a Y-axis misalignment. This needs to be verified for other settings for the moveable jaws as well.

#### Rotational beam stability

The rotational variation test results showed that the machine is stable with rotation and the subsequent constancy in the output and energy was observed. The energy of the beam changes the shape of the profile of linac. Higher energy beams are more forward directed and have lowered shoulders with respect to the center. Placing the MVCT detector array in a position such that it is aligned to the center of the jaws ensures that the optimum detector response will be obtained during a TomoImage scan, maximizing image quality with regard to noise.

**Figure 8:**
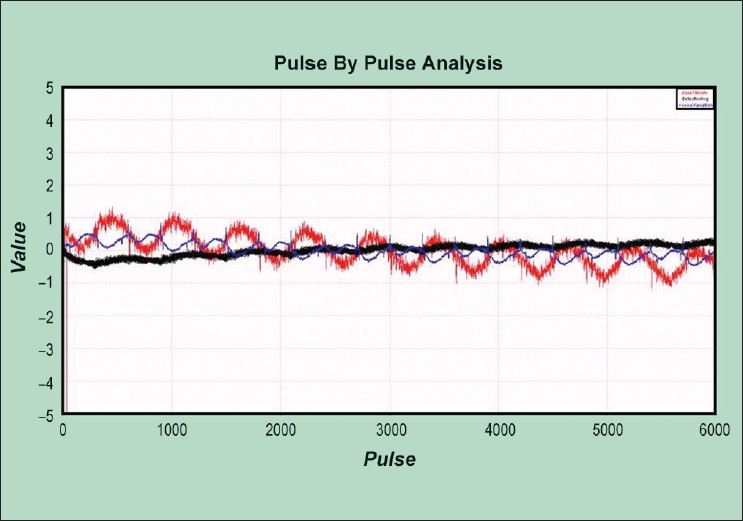
The test performed for rotational beam stability shows the comparison of the measured average profile with the reference data. The measured data was in good agreement with the reference data

[Figure 8] shows the comparison of measured average profile with the reference data. The measured data was in good agreement with the reference data. The results showed an acceptable tilt of 1-2%. The energy constancy was found to be 99% (range 99-100.5) with maximum gamma as 0.4.

### Secondary beam alignment

#### MLC Center-of-Rotation (COR) twist

**Figure 9:**
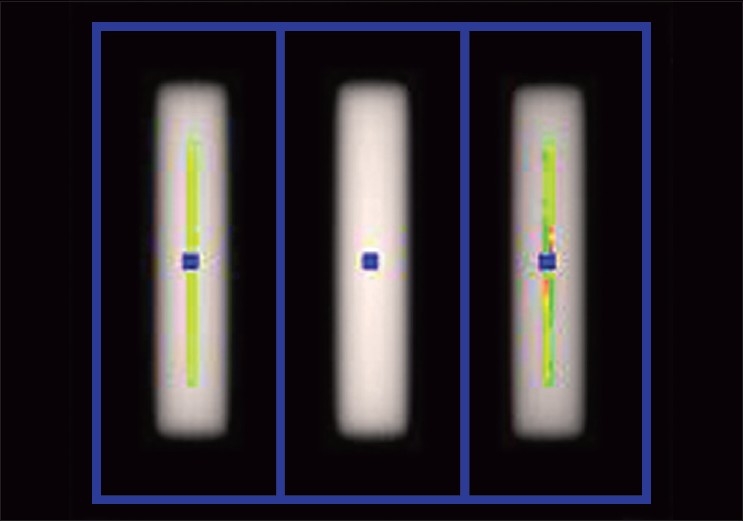
The dose image that resulted from a double exposure gantry rotation, in which leaves 27 and 28, and leaves 32 and 33 opened sequentially. MLC COR twist test was found satisfactory with the an MLC center offset of 0.48 mm

[Figure 9] shows the dose image that resulted from a double exposure gantry rotation in which leaves 27 and 28 and leaves 32 and 33 opened sequentially. MLC COR twist test was found satisfactory with MLC Center offset of 0.48mm which was well within acceptable tolerance (1.5mm). The MLC twist angle was found to be 0.003°. Difference between left and right separations was 0.96mm. This proved the best MLC stability and alignment during the gantry rotation.

#### Field center versus jaw setting

**Figure 10:**
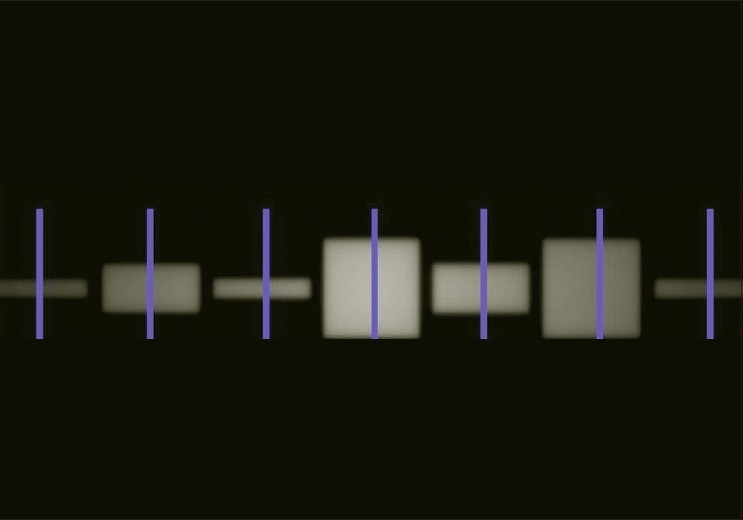
The dose image that resulted from exposures to an EDR2 film when performing the field center versus jaw setting test. In this test a series of leaves were opened for the clinical jaw setting of 1 cm, 2.5 cm, and 5.0 cm. Both the jaws moved symmetrically if the centers of the dose blocks were the same. The results of this test verified that the jaws close symmetrically and the centers for different field sizes were aligned

[Figure 10] shows the dose image that resulted from exposures to an EDR2 film in which a series of leaves were opened for the clinical jaw setting of 1cm, 2.5cm and 5.0cm. Both the jaws moved symmetrically if the centers of the dose blocks are the same. Field Center versus Jaw Setting Test verified that jaws close symmetrically and the centers for different field sizes were aligned. The maximum and mean center difference was found to be 0.066 mm and 0.027 mm respectively. This proved the satisfactory jaw alignment during the gantry rotation.

### Water tank measurements (profiles and depth doses)

#### Transverse and longitudinal beam profiles

**Figure 11:**
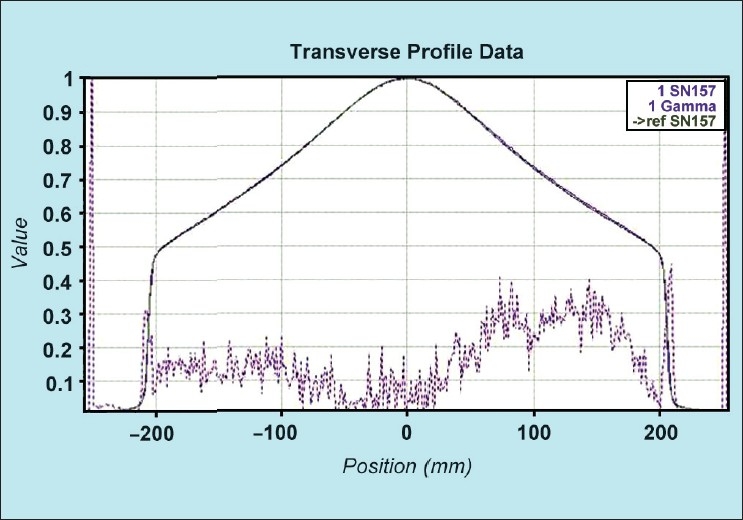
The comparison of measured and reference transverse profiles for 5 cm jaw width

[Figure 11] shows the comparison of transverse profiles (measured and factory data) for 5 cm field width. The percentage variation between full width quarter maximum (FWQM) from measured and reference profile of 50 mm width was −0.11. Similarly 25 mm and 10 field widths, this variation was found to be −0.08 and −0.07 respectively. The test was found to be passed for Gamma criteria of 2.0% and 1 mm for all three jaw widths. The absence of flattening filter in the tomotherapy machine lowers the head scatter contribution which is not the case in conventional linacs. Hence the shape of the transverse profile is not flat as compared to the conventional linac. This profile is modulated by the MLC. Hence the FWQM defines the field size.

**Figure 12:**
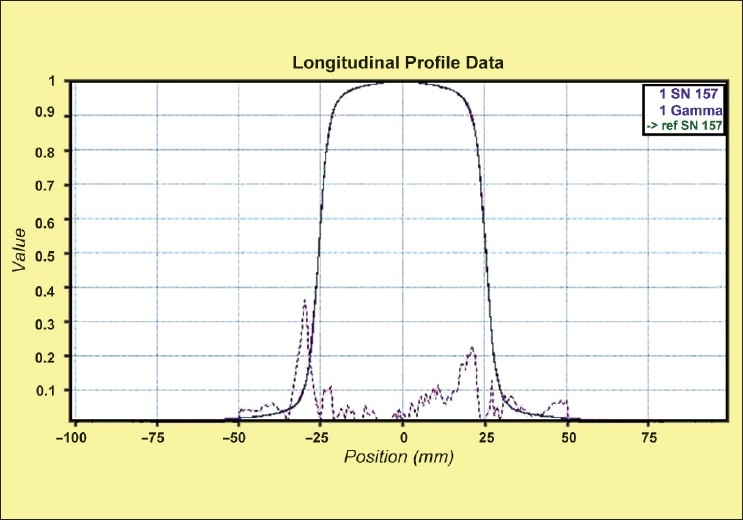
The comparison of measured and reference longitudinal profiles for 5 cm jaw width

[Figure 12] shows the comparison of longitudinal profiles (measured and factory data) for 5 cm field jaw width. For longitudinal profiles, the percentage variation between full width half maximum (FWHM) from measured and reference profile of 50 mm width was 0.06. The gamma was passed for 2.0% and 0.5 mm. Similarly for 25 mm and 10 field widths, this variation was found to be 0.09 (Gamma of 2.0% and 0.25 mm) and −0.85 (Gamma of 2.0% and 0.1 mm) respectively. The test was found to be passed for Gamma criteria of 2.0% and 1 mm for all three jaw widths. Thus both the transverse and longitudinal profiles were in good agreement with the “Gold” standard for 50, 25 and 10 mm field Y-axis. The static longitudinal (Y-profile) is smeared out by the helical tomotherapy jaws. Since the profile is taken only for a maximum 5 cm jaw width, the shape of the profile looks apparently a flat though in reality is not the case. Here the concept of FWHM holds good for longitudinal profile. This profile is an important parameter for helical tomotherapy because the dose shape is continuously superimposed with slight offsets as the patient moves into and away from the treatment beam. Thus classical measures of the profile parameters (flatness and symmetry) are not relevant.

#### Central axis depth dose

**Figure 13:**
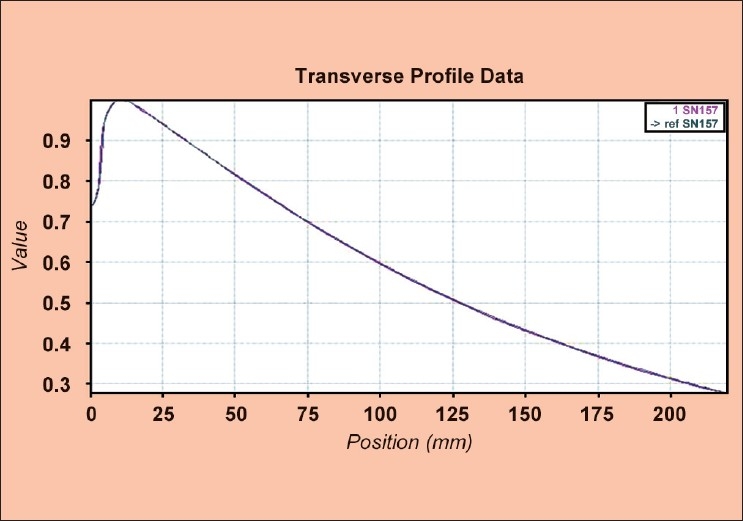
CADD profiles for 5 cm jaw width

[Figure 13] shows the CADD profiles for 5 cm. For 5 cm jaw width, the CADD measurements showed the Dmax of 1.14 cm. The percentage variation between measured and factory ratio (D20/D10) was −0.19. Similarly for 2.5 cm and 1.0 cm jaw widths, the percentage variation between the ratios was found to be −0.38 and −0.11 respectively. The measured CADD ratio (20/10) was 0.52 and found in good agreement with the factory data.

The central axis depth dose variation is typically rather greater than that of conventional 6 MV machine because of the reduced source to axis distance.

### Dose rate

Tomotherapy machine do not work on a monitor unit-based system, but operate more like a cobalt unit. Output is therefore calibrated in terms of a reference dose rate, measured in units of cGy per minute rather than the conventional cGy per monitor unit. The dose rate (output) at 85cm SAD (1.5cm buildup) was found to be 886 cGy/ min compared to 890 cGy/min (<1%). This value was then used to set the correct calibration for the dose 1 and dose 2 monitor units (MU) displays at the control console monitor.

### Intensity modulated radiotherapy verification

**Figure 14:**
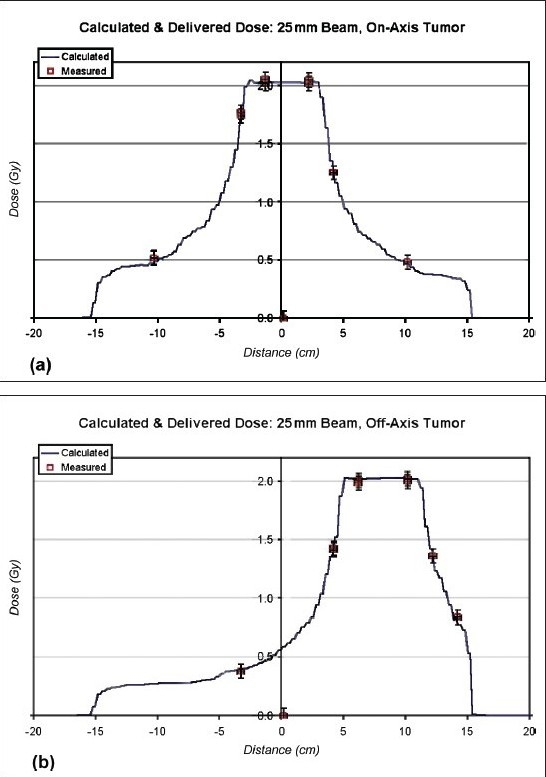
(a) IMRT plan verification with 2.5 cm jaw width for on-axis tumor. (b) IMRT plan verification with 2.5 cm jaw width for off-axis tumor

Various IMRT plans were run to measure the doses on the central axis and the off-axis. [Figure 14a and Figure 14b)] shows a 2.5 cm jaw width IMRT plan verification for on-axis and at off-axis, respectively. During IMRT verification, the variation between the measured and calculated dose for a particular plan at the central axis and off-axis was found to be within 2% (2% dose and 1mm in position, respectively). Thus for a 1 cm, 2.5cm, and 5 cm jaw width, the entire IMRT verification was found to be acceptable. This test revealed the precision of the MLC and the calculation accuracy of the beam model.

The IMRT dose calculation algorithm assumes that the 64 leaves in the MLC are divided evenly about the isocenter (i.e., there 32 leaves on one side of the isocenter and 32 on the other). It also assumes that the leaves are parallel to the Y-axis. Significant deviations of the machine from either of these assumptions can cause discrepancies in IMRT delivery, so it is important to ensure that these aspects of beam geometry are within tolerance. The jaw width of 2.5 cm is most often used in IMRT planning. Hence it is important to verify at least the 2.5 cm jaw width. However, we verified the other two as well (1cm and 5 cm). Since the jaw width is less, the treatment time is relatively longer.

### MVCT dose

The measured dose during MVCT procedure (fifteen slices) was found to be 2.57 cGy (specifications <4 cGy).

## Conclusion

The first clinical Helical Tomotherapy Hi-Art II machine was installed at our center. The dosimetric validation tests for this machine were presented. All the dosimetric parameters were compared with the factory data and thus it was acceptable. The exact findings of our results were reported along with the specifications from the manufacturer. The Helical Tomotherapy system is mechanically stable with a great synchronization between gantry, MLC, and the couch, and proves to be a novel approach for IMRT treatment. However, the machine should be continuously monitored for any fluctuations in dose rate (output). Hence it is recommended that the static output of the machine be measured on a daily basis in addition to the ratio (D20/D10).
